# Lecanemab, aducanumab, and donanemab bind the same human Alzheimer disease Aβ aggregates at different epitopes

**DOI:** 10.1002/alz70855_099914

**Published:** 2025-12-23

**Authors:** Andrew M Stern, Anna Francis, Angela Meunier, Amirah K Anderson, Elizabeth L Hennessey, Michael B Miller, Cynthia A Lemere, Dennis J. Selkoe

**Affiliations:** ^1^ Ann Romney Center for Neurologic Diseases, Brigham and Women's Hospital, Harvard Medical School, Boston, MA, USA; ^2^ Ann Romney Center for Neurologic Diseases, Brigham and Women's Hospital, Harvard Medical School, Boston, MA, USA; ^3^ Brigham and Women's Hospital, Boston, MA, USA

## Abstract

**Background:**

Lecanemab, donanemab, and aducanumab all bind to aggregates of Aβ and all clear amyloid PET in clinical trials, but they are proposed to bind different forms of Aβ and cause amyloid related imaging abnormality (ARIA) at different rates. We tested the binding properties of in‐house recombinant versions of these antibodies to soluble (aqueous), detergent‐insoluble, amyloid plaque‐enriched, and cerebral amyloid angiopathy (CAA)‐enriched extracts of human AD brain.

**Methods:**

We developed a new immunoprecipitation‐ELISA method for measuring antibody equilibrium binding affinity (K_D_) and total antigen availability (B_max_) to Aβ40‐rich aggregates from leptomeninges, likely principally CAA, and to Aβ42‐rich aggregates from grey matter, likely principally plaque, in aqueous and *N*‐lauryl‐sarcosine‐insoluble fractions. We used linear mixed models to measure differences and correlations between antibody K_D_, B_max_, and ratios thereof, and the effects of *APOE* genotype and sex across 18 AD cases.

**Results:**

The B_max_ was highly correlated between antibodies (*r* > 0.9) for all Aβ aggregate extracts. Lecanemab did not exhibit a preference for aqueous Aβ (“protofibrils”) compared to aducanumab. Aducanumab, which causes the most ARIA, did not consistently exhibit greater preference for CAA Aβ compared to lecanemab or donanemab, which cause less ARIA.

**Conclusions:**

Within the limitations of measuring *in vitro* equilibrium binding activity of in‐house recombinant antibodies to aqueously extracted Aβ from human *postmortem* brain tissue, we could not discern a population of Aβ aggregates (*e.g*. protofibrils) accessible to one antibody but not the other two. Antibody preference for CAA Aβ over plaque Aβ does not explain differences in clinical trial ARIA rates.

